# ICU Delirium as a Failure of Predictive Synchronization: A Two-Agent Active Inference Model

**DOI:** 10.3390/e28060702

**Published:** 2026-06-17

**Authors:** Luca M. Possati

**Affiliations:** Faculty of Behavioural, Management and Social Sciences, Philosophy Section, University of Twente, Drienerlolaan 5, 7522 NB Enschede, The Netherlands; l.m.possati@utwente.nl

**Keywords:** ICU delirium, active inference, free-energy principle, design, human-technology interaction

## Abstract

This paper presents a computational model of delirium in the Intensive Care Unit (ICU), in which delirium is defined as the endpoint of a self-reinforcing cycle of predictive failure between two bidirectionally coupled agents: the patient and the ICU room environment. Drawing on the active inference framework and the free energy principle, the paper proposes that delirium is not a property of the patient in isolation but a relational phenomenon that emerges when the environment persistently fails to predict the patient’s internal state. This failure triggers a causal feedback mechanism in which desynchronization pressure progressively sharpens the patient’s prior beliefs—implementing precision rigidity in the correct active inference sense: not a brain overwhelmed by noise but a brain locked into a state that incoming observations can no longer update. The model is implemented as a two-agent POMDP in which both agents maintain generative models and continuously attempt to predict each other’s states. The room agent (R)—understood as the environment-side sensing–inference–actuation loop, whether instantiated by clinical staff or by an automated monitoring system—infers the patient (P)’s latent parameters $\left(\theta_{\mathrm{cog}},\theta_{\mathrm{emo}}\right)$ over time and builds a progressively personalized generative model of the patient. Synchronization is operationalized via two commensurable directional surprisal metrics: $S_{R\to P}=-\ln Q_{R}\left(s^{*}\right)$, the room’s surprisal at the patient’s true state, and $S_{P\to R}=-\ln P\left(o_{R}|Q_{P}\right)$, the patient’s surprisal at the room’s observations. A systematic ablation study across four model variants shows that room inference is the architectural component necessary to reproduce the synchronization–delirium relationship: when the room infers, the association between synchronization and declared delirium is strong and stable, whereas a non-inferring room collapses to ceiling delirium rates and a weak association. θ learning and the prior-sharpening feedback do not increase the strength of this association; instead they shape the phenotypic gradient, reducing ceiling effects in vulnerable phenotypes and amplifying the separation between them. The model is presented as a computational hypothesis generator rather than a calibrated clinical predictor, and its implications for ICU design are discussed.

## 1. Introduction

Delirium is the most prevalent acute neuropsychiatric syndrome in the Intensive Care Unit (ICU), affecting 40–80% of mechanically ventilated patients and carrying significant consequences for mortality, length of stay, and long-term cognitive outcomes [1,2]. Despite its clinical burden, the mechanistic pathway linking the ICU sensory environment to delirium onset remains poorly understood. Non-pharmacological interventions—light management, acoustic control, sleep protection—are recommended by international guidelines but are rarely implemented on a principled, biologically grounded basis.

The standard account treats delirium as a consequence of excessive stimulation or systemic illness overwhelming the patient’s cognitive resources. The present paper proposes a different explanation. The problem is not that the environment sends too much—it is that the environment and the patient stop understanding each other. The paper proposes a computational two-agent POMDP model grounded in the active inference framework [3,4,5] to explain the onset of delirium in the ICU. The central hypothesis is that delirium arises from the failure of predictive synchronization between the patient and the sensory environment of the ICU room.

Under the active inference framework [6,7,8,9,10,11,12], the brain continuously updates its internal model of the world by comparing predictions to incoming observations. This works as long as the environment generates sensory patterns the brain can track. In the ICU, however, the environment often fails to do this: lights, sounds, and clinical interventions are not calibrated to the patient’s current internal state. When this mismatch persists, the patient’s brain responds not by becoming confused but by becoming increasingly certain—locking its prior beliefs into a fixed state that no observation can update. This can be called *precision rigidity*. The patient is not overwhelmed. The patient is locked in beliefs that they consider extremely certain. Therefore, delirium is a bidirectional collapse of predictive capacity. It is not a problem of the patient, and it is not a problem of the environment. It is a failure of the coupling between the two.

Critically, this process is self-reinforcing. A locked patient generates physiological signals that the environment’s current model predicts increasingly poorly—not because the signals are inherently less predictable but because the environment’s model has become misaligned with the patient’s state, so prediction error grows. This causes the environment to produce even less calibrated stimuli, which deepens the lock. Delirium is the clinical endpoint of this spiral—not a static deficit but a dynamic breakdown of the coupling between patient and environment. This hypothesis develops some ideas already presented in [13].

The paper formalizes and tests this hypothesis computationally. We model the ICU as two bidirectionally coupled agents—patient and room—each maintaining a generative model and continuously attempting to predict the other. The patient agent (Section 3.3.1) is a compact POMDP whose hidden state captures cognitive precision, emotional precision, and circadian alignment, and whose constrained action repertoire reflects an intubated individual; the room agent (Section 3.3.4) implements hierarchical active inference, building a progressively personalized model of the patient over time. The two models are deliberately asymmetric—the patient infers and acts within a narrow margin, the room infers and acts across light and sound—and the points at which they converge and diverge are made explicit in Section 3.3. The causal mechanism is implemented explicitly: persistent failure of the room to predict the patient triggers prior sharpening in the patient, progressively reducing the influence of incoming observations until the patient’s belief updating stalls. We then test whether this architecture produces the qualitative patterns predicted by the hypothesis—phenotypic gradient in delirium risk, room-side signal preceding patient-side confusion, each architectural component contributing independently—and whether those patterns are stable across seeds, parameter values, and model variants.

The model presented in this paper is intended as a computational hypothesis generator, not as a calibrated clinical predictor. Its parameters were specified from neurobiological priors rather than patient data. Accordingly, its numerical outputs (delirium rates, surprisal values, correlation coefficients) should not be read as estimates of clinical quantities. They serve a different purpose: to verify that the proposed causal architecture produces qualitative patterns—a phenotypic gradient, a room-side signal that precedes patient-side confusion, an association between synchronization and delirium—that are internally consistent with the hypothesis and coherent with established clinical knowledge.

The scope is restricted to the sensory–environmental component of delirium; systemic contributors are represented as static phenotypic parameters. So the question is, *given that a patient has a certain vulnerability profile, how does the coupling between that patient and the ICU sensory environment contribute to delirium onset?* Validation against real physiological data—EEG, heart rate variability, continuous behavioral monitoring—remains the necessary and explicit next step.

From a broader psychological and philosophical perspective, this research is particularly compelling for two main reasons. The first concerns the environmental dimension—specifically, the intimate connection between the patient’s psychological state and the surrounding environment [14,15]. The model demonstrates that, in ICU delirium, the sensory environment is not a passive backdrop: together with the inferential and actuating system that governs it—whether clinical staff or an automated monitoring layer—it functions as an active inference agent in the model, one that participates directly in shaping the patient’s trajectory. The ICU context is highly distinctive: the patient is physically tethered to machines and deeply immersed in a technological environment upon which their survival literally depends. The relationship with technology—what the literature calls “technical mediation” [16,17]—is therefore crucial [13]. The simulation is consistent with this view in a specific sense: when the environment-side loop maintains and updates a model of the patient, the coupling reproduces the expected phenotypic gradient and synchronization–delirium association, whereas a non-inferring environment does not (Section 3.6). This supports the claim that the environment functions as a participant in the patient’s trajectory rather than a passive backdrop; it does not, by itself, validate the mechanism empirically.

## 2. Clinical Background and Theoretical Motivation

This section offers a selective overview of the recent literature on ICU delirium in order to situate the computational model developed in this paper. Rather than providing a systematic review, it focuses on the themes most relevant to the present framework: risk factors for delirium onset, delirium subtypes, and the main approaches to prevention and management. Particular attention is given to findings that motivate the model’s emphasis on patient vulnerability, environmental stressors, and sensory modulation.

The recent literature describes ICU delirium as a multifactorial condition emerging from the interaction between baseline vulnerability, acute illness, and iatrogenic or environmental stressors associated with intensive care [18,19]. Predisposing factors repeatedly identified include older age, pre-existing cognitive impairment, comorbidity burden, visual or hearing impairment, malnutrition, and depression; acute precipitants include infection, sepsis, metabolic derangements, hypoxia, and other severe physiological disturbances [19,20,21]. Delirium risk is further increased by common features of ICU care itself, including mechanical ventilation, deep or prolonged sedation, benzodiazepines, opioids, physical restraints, invasive devices, immobility, and sleep disruption [19,20]. Taken together, these studies support the view that ICU delirium is better understood as the product of converging vulnerabilities and exposures than as the consequence of a single linear cause [22,23].

The literature consistently distinguishes three motor subtypes of ICU delirium: hypoactive, hyperactive, and mixed [24,25]. Among these, hypoactive delirium appears to be the most common, yet it is also the subtype most likely to be missed in routine care because of its reduced behavioral salience; by contrast, hyperactive delirium is more readily recognized because of overt agitation and disruptive behavior [19,25].

Regarding prevention and management, the literature emphasizes early recognition, systematic assessment, and prompt treatment of underlying causes, together with multicomponent non-pharmacological strategies [18,26]. In particular, approaches organized around the ABCDEF bundle, early mobilization, and family participation appear more promising than isolated interventions, although the certainty of evidence remains variable [18,19]. This is especially relevant for the present framework because recent work on sensory-based and auditory interventions suggests that modulation of the patient’s sensory environment may reduce confusion and delirium risk, particularly when familiar and meaningful cues are used [18,27]. By contrast, current evidence does not support the routine use of antipsychotics as a general strategy for delirium prevention or standard treatment; their role remains limited to selected situations of severe agitation or immediate risk [19,28].

Several limitations in the current literature are directly relevant to the present model and its motivations. First, evidence for non-pharmacological and multicomponent interventions remains heterogeneous, with important inconsistencies in effect estimates across reviews and intervention types [18]. Second, the field still lacks sufficient standardization in intervention design, outcome definition, and reporting, which makes comparisons across studies difficult and weakens cumulative inference [18,28]. Third, even where bundles such as ABCDEF appear promising, implementation in routine ICU practice remains a major unresolved challenge [18]. Finally, although the long-term burden of ICU delirium on cognition, daily functioning, and sleep is increasingly recognized, long-term and patient-centered outcomes remain less developed than in-hospital endpoints in the intervention literature [29].

These limitations point to a deeper theoretical problem: the absence of a mechanistic framework capable of specifying which variables matter, why they matter, and for whom they matter. In this respect, the heterogeneity documented in the intervention literature should not be read only as a methodological weakness but also as evidence that the field lacks a sufficiently explicit model linking patient-specific vulnerability to concrete inputs [18,27]. This paper aims to help fill this gap by focusing on environmental variables—this does not mean considering these variables as the only ones.

The model proposed in this paper is intended to address this gap through the predictive synchronization hypothesis. Rather than introducing another intervention bundle, it formalizes a mechanistic account of how the ICU environment may contribute to, amplify, or mitigate delirium. On this view, environmental interventions are expected to differ in effectiveness depending on how they modify the dynamic relation between patient state, sensory input, and adaptive regulation. The model is therefore designed to explain why hypoactive delirium is often missed, why patient groups may respond differently to apparently similar interventions, and why trajectories of patient–environment synchronization may constitute a more sensitive outcome than delirium incidence alone.

A further strand of work, directly relevant to the present framework, concerns active inference in multi-agent settings, where two or more agents each maintain a generative model of the other and coordination emerges from—or breaks down through—their mutual predictability. Refs. [30,31] formalize communication between two agents as a process of generalized synchrony, in which each agent comes to predict the other’s dynamics; ref. [6] extend this to socially shared expectations and conformity. Ref. [32] further develop this approach. The notion of (mis)alignment between coupled generative models is central to this literature: synchronization is maintained when agents successfully predict one another, and degrades when their models diverge. The present model applies this two-agent logic to a clinical setting in which the two agents are deliberately asymmetric—a patient with a constrained motor repertoire and an environment-side inferential loop—and asks what happens to the patient when the coupling fails. To our knowledge, ICU delirium has not previously been framed as a failure of predictive synchronization between coupled active inference agents.

## 3. The Predictive Synchronization Hypothesis

### 3.1. Delirium as a Failure of Predictive Synchronization

The central theoretical claim of this paper departs from standard accounts of ICU delirium in a precise way. Standard accounts treat delirium as a consequence of excessive sensory stimulation or systemic inflammation overwhelming the patient’s cognitive resources [33,34]. The present model proposes instead that delirium is a *relational phenomenon*—not a property of the patient in isolation but of the coupling between the patient and the environment.

The formal basis for this claim is the active inference framework. Under active inference, the brain is not a passive receiver of sensory signals but a probabilistic inference engine that continuously minimizes *variational free energy* (VFE) by updating its internal generative model against incoming observations [4]. Delirium, on this account, corresponds to a pathological elevation of precision (in probability theory, the precision of a distribution is simply the inverse of its variance) on prior beliefs that prevents sensory evidence from updating the generative model. The patient’s internal model becomes decoupled from the world.

Two clarifications are needed about the status of this claim. First, the identification of delirium with pathologically elevated prior precision is a modeling hypothesis, not an established physiological fact. It is motivated—not proven—by several convergent lines of evidence: the dependence of sensory precision on cholinergic thalamocortical gating, which is disrupted in delirium [34,35]; the neuroinflammatory changes associated with delirium that plausibly alter neuromodulatory tone [33]; and the predictive-coding account of aberrant inference under imbalanced precision [36]. The present model takes this hypothesis as its starting point and asks what follows from it dynamically, rather than deriving it from first principles. Alternative mechanistic readings of delirium exist—for example, an internal loop that runs without sensory correction, or the dysfunction of a specific neural population—and the present account is not incompatible with them; it isolates one pathway (precision imbalance in the patient–environment coupling) and examines it in isolation. Second, elevated prior precision is not specific to delirium: the same computational signature appears, with different content and clinical surface, in conditions such as hallucination [36]. What the model claims is therefore not that precision rigidity is sufficient for delirium but that, *given a vulnerable patient embedded in a poorly synchronized environment*, it is a plausible and sufficient-within-the-model pathway to the hypoactive presentation. Establishing its specificity and its physiological signature against competing mechanisms is an empirical question, to be settled by the prospective monitoring study outlined in Section 3.5.6, not by the present simulation.

**Definition** **1** (Precision rigidity)**.** Precision is the inverse variance of a probability distribution. When the precision of prior beliefs becomes pathologically elevated, the posterior distribution is insensitive to prediction errors: $Q^{*}\left(s\right)\approx Q_{\mathrm{prior}}\left(s\right)$ regardless of the observation o. We call this condition precision rigidity.

It is important to clarify what precision rigidity is *not*. It is not a brain that stops receiving signals or that is overwhelmed by noise. It is a brain that receives signals normally but discounts them because its prior beliefs are held with such high confidence that no observation can shift them. The patient is not confused by the environment—the patient is *locked* into an internal model that the environment can no longer update. This distinction has a direct consequence for how the causal feedback mechanism of delirium is implemented as described in Section 3.4.7.

The model’s central hypothesis extends this account by specifying the *cause* of precision rigidity. According to [30], an agent is *synchronized* with another agent when it can reliably predict the other’s states. From this point of view, ICU delirium emerges from a bidirectional causal loop: the environment (room) fails to predict the patient’s internal state (room’s surprisal: $S_{R\to P}$ high); this persistent failure progressively sharpens the patient’s prior beliefs to the point where incoming observations can no longer update them (precision rigidity); and the resulting rigidity prevents the patient from predicting the environment’s observations (patient’s surprisal: $S_{P\to R}$ high). Delirium is the clinical manifestation of this self-reinforcing cycle in which de-synchronization causes precision rigidity, which further impedes the patient’s capacity to re-synchronize with the environment.

This account maps directly onto the predictive-coding reading of inference. Under predictive coding, perception is the adoption of a hypothesis that best explains the current sensory input, weighted by prior expectations; priors are compared against incoming signals, and prediction errors are computed [36]. The relative precision of priors and sensory inputs determines which dominates: when priors are more precise than sensory inputs, they dominate inference, and prediction errors are effectively ignored; when prediction errors are relatively more precise, they drive belief updating. Precision rigidity is the limiting case of the first regime. As desynchronization pressure sharpens the patient’s prior (Section 3.4.7, Equation (20)), the prior becomes so precise that incoming prediction errors are discounted regardless of content, and belief updating stalls. The same mechanism that ref. [36] invokes for inordinately precise priors—there to explain hallucinations—here produces the opposite phenotype: not a percept without a stimulus but a state locked against stimuli. In both cases the common cause is a pathological imbalance of precision between priors and prediction errors; what differs is the direction and the clinical surface (see also, for predictive processing, ref. [37]).

A note on symmetry and the limits of the simulation. The hypothesis is formulated as bidirectional, and the model implements both directions. However, the two directions are not equally informative in the current simulation: $S_{R\to P}$ differentiates phenotypes substantially more than $S_{P\to R}$, and the causal feedback operates primarily through the room-side signal $S_{R\to P}$ (the room’s surprisal at the patient’s true state, defined in Section 3.2).

This asymmetry must be interpreted with caution. A clarification is needed here to avoid a misunderstanding. The true hidden state $s^{*}$ is *not* available to the room agent during inference: the room never observes $s^{*}$ and forms its belief $Q_{R}\left(s\right)$ only from the physiological signals $o_{R}$, which is precisely why a prediction error can exist. The true state $s^{*}$ enters only at the level of the simulator, as the quantity against which we *evaluate* the room’s belief: $S_{R\to P}=-\ln Q_{R}\left(s^{*}\right)$ measures how much probability mass the room’s (uninformed) belief happens to place on the state that is in fact true. The room can therefore be wrong about the patient even though $s^{*}$ appears in the metric, because $s^{*}$ is used by us to score the room, not by the room to infer. The consequence is that $s^{*}$ exists in the simulator but has no direct clinical equivalent: any clinical implementation would have to estimate $S_{R\to P}$ from observable physiology.

The apparent dominance of the room-side signal may therefore reflect, at least in part, a structural privilege of the metric rather than a property of the underlying dynamics. Whether the same asymmetry holds when both signals are estimated from observable physiological proxies—as any clinical implementation would require—is an empirical question that the present model cannot answer.

What the simulation does establish is the theoretical architecture: both directions of prediction failure are necessary for declared delirium, and the room’s failure to track the patient is the variable through which the causal feedback operates. The clinical implication—that environment-side monitoring may provide earlier warning than patient-side monitoring—is a falsifiable prediction, not a finding. Testing it requires prospective ICU data in which both signals are estimated simultaneously from continuous physiological monitoring.

Now, the hypothesis has two immediate consequences. First, it is not testable with a single-agent model—it requires two agents, each maintaining a generative model, whose mutual predictive capacity can be independently measured. Second, it predicts that delirium should be preceded by a period of measurable de-synchronization on the room side, offering a potential early-warning signal.

### 3.2. Synchronization Is Operationalized as Surprisal

We quantify the predictive capacity of each agent via directional surprisal metrics that are mathematically commensurable: both are negative log-probabilities of an observation under a belief.

Let $s_{P}^{*(t)}$ denote the patient’s true hidden state at cycle *t*—the actual configuration $\left(\gamma_{cog},\gamma_{emo},circ\right)$ the patient occupies, one of the 48 discrete hidden states defined in Section 3.3.1—and let $Q_{R}^{\left(t\right)}\left(s\right)$ denote the room’s belief over those states. The room’s surprisal at the patient’s true state is

(1) $$S_{R\to P}^{\left(t\right)}=-\ln Q_{R}^{\left(t\right)}\;\left(s_{P}^{*(t)}\right).$$

When $S_{R\to P}$ is low, the room’s model of the patient is accurate; when it is high, the room has lost track of the patient’s internal state.

The patient’s surprisal at the room’s observation is

(2) $$S_{P\to R}^{\left(t\right)}=-\ln\;E_{Q_{P}^{\left(t\right)}}\;\left[\;P(o_{R}^{\left(t\right)}|s)\;\right]=-\ln\sum_{s}Q_{P}^{\left(t\right)}\left(s\right)\;P\;\left(o_{R}^{\left(t\right)}|s\right).$$

where the three quantities have the following interpretations. Here $P(o_{R}|s)$ is the room’s likelihood, implemented as the matrix $A_{R}$ with entries $A_{R}\left[o_{R},s\right]=P\left(o_{R}|s\right)$; we write it as $A_{R}$ wherever the matrix form is needed for the inference equations, and as $P(o_{R}|s)$ where the probabilistic meaning is what matters.

$o_{R}^{\left(t\right)}$ is the room’s actual observation at cycle *t*: one of the 24 physiological signals in $O_{R}$, encoding the patient’s current heart rate level, psychomotor activity, and EEG state as registered by the ICU sensors.

$Q_{P}^{\left(t\right)}$ is the patient’s current belief over its own 48 hidden states: a categorical distribution whose probability mass function, evaluated at a state $s=(\gamma_{cog},\gamma_{emo},circ)$—the triple of cognitive precision, emotional precision, and circadian alignment defined in full in Section 3.3.1—returns the scalar $Q_{P}^{\left(t\right)}\left(s\right)$, the probability the patient assigns at cycle *t* to being in that state. These scalars sum to one over the 48 states.

$A_{R}\left[o_{R},s\right]=P\left(o_{R}|s\right)$ is one entry of the room’s likelihood matrix, encoding the probability that the room would register observation $o_{R}$ if the patient’s hidden state were *s*. We emphasize that $A_{R}$ belongs to the *room* agent (the environment-side inferential loop defined in Section 3.3.4), not to the patient: it is the model the environment uses to map the patient’s internal states to the physiological signals it observes. There are not two models inside the patient’s brain. There are two agents—patient and room—each maintaining a single generative model of the other; $A_{P}$ is the patient’s likelihood (how room actions produce the patient’s sensations), and $A_{R}$ is the room’s likelihood (how patient states produce physiological signals). The quantity $A_{R}$ is high when state *s* would, with high probability, generate observation $o_{R}$, and low when $o_{R}$ is unlikely given *s*.

The summation $\sum_{s}Q_{P}^{\left(t\right)}\left(s\right)\cdot A_{R}\left[o_{R}^{\left(t\right)},s\right]$ combines these three quantities into a single number: the marginal probability of the room’s actual observation under the patient’s current belief. For each of the 48 hidden states (the $N_{S}=4\times 4\times 3=48$ combinations of $\left(\gamma_{cog},\gamma_{emo},circ\right)$ defined in Section 3.3.1), it takes the probability that the room would generate $o_{R}$ from that state and weights it by how strongly the patient currently believes it is in that state. Summing across all states yields the overall probability that the patient’s generative model would have predicted the room’s actual output. Taking the negative logarithm converts this probability into a surprisal in nats: when the patient’s model predicted the room’s output well, $S_{P\to R}$ is low; when the room generated something the patient did not expect, $S_{P\to R}$ is high. This quantity answers a precise question: *given what the patient believes about itself, how probable was the observation the room actually generated?*

The result is commensurable with $S_{R\to P}^{\left(t\right)}=-\ln Q_{R}^{\left(t\right)}\left(s_{P}^{*(t)}\right)$: both quantities are negative log-probabilities of an observed event under a belief distribution, one measuring the room’s surprise at the patient’s true state, the other measuring the patient’s surprise at the room’s actual observation. They differ in one structural respect: $S_{R\to P}$ is anchored to the patient’s true hidden state $s^{*}$, which is accessible in simulation but not directly observable in clinical settings; $S_{P\to R}$ is anchored to the room’s actual observation $o_{R}$, which is measurable from physiological sensors. This asymmetry in clinical observability does not affect the mathematical commensurability of the two metrics.

Previous implementations proxied $S_{P\to R}$ with the patient’s variational free energy $F_{P}$. This is not commensurable with $S_{R\to P}$. At the variational optimum and in the absence of additional constraints on the approximate posterior, the free energy equals the surprisal; when the posterior is constrained, the free energy exceeds the surprisal by a non-negative divergence term, $F_{P}=-\ln P\left(o_{P}\right)+\mathrm{KL}\;\left[Q_{P}\;∥\;P\left(s|o_{P}\right)\right]$. Using $F_{P}$ as a proxy therefore adds this divergence term to the quantity of interest so that $F_{P}$ no longer measures the patient’s predictive failure alone but mixes it with the suboptimality of the patient’s posterior. Since $S_{R\to P}$ contains no such term, the two were not on the same footing. The current implementation uses the commensurable surprisal form in Equation (2) throughout, in which both directions are pure negative log-probabilities.

The composite synchronization index is

(3) $${\mathrm{SI}}^{\left(t\right)}=\frac{1}{2}\left(1-\min\;\left(\frac{S_{R\to P}^{\left(t\right)}}{S_{\max}},1\right)+1-\min\;\left(\frac{S_{P\to R}^{\left(t\right)}}{S_{\max}},1\right)\right)\in \left[0,1\right],$$

with $S_{\max}=6.0$ nats. SI=1 corresponds to perfect bidirectional synchronization, and SI=0 to complete failure of mutual prediction.

### 3.3. Agent Architecture

The model consists of two bidirectionally coupled POMDP agents. Each agent is fully characterized by a likelihood matrix *A*, a transition matrix *B*, a preference vector *C*, and an initial prior *D*. All distributions are categorical. One decision cycle corresponds to approximately 60 s of real ICU time.

A word on the discrete formulation. The physiological and psychological processes underlying delirium are, at the substrate level, continuous in both state and time, and a fully faithful model would represent them as such (for example, as a continuous-state, continuous-time active inference process). We deliberately adopt a discrete-state, discrete-time POMDP for three reasons. First is tractability: discrete-state active inference makes variational state estimation, hierarchical parameter learning, and expected-free-energy policy selection exactly computable without sampling approximations, which is what allows the ablation and robustness analyses of Section 3.5 to be run exhaustively. Second is precedent: discrete-state POMDPs are the standard formalism for active inference models of cognition and behavior ([4]), so the discrete choice keeps the model comparable to that literature. Third is level of claim: the conclusions we draw are qualitative and ordinal—the direction of the phenotypic gradient and the sign of the synchronization–delirium association—and these do not depend on fine numerical resolution of the state space. The discretization (48 hidden states, a 10×10 parameter grid, one cycle ≈60 s) is therefore a coarse but deliberate approximation of a continuous substrate. Whether a continuous-state formulation would preserve, sharpen, or qualify these conclusions is a question of model-form robustness, which we return to in Section 3.8 and leave as an explicit extension.

#### 3.3.1. The Patient

The patient is not represented in their full clinical complexity but through three essential dimensions: how cognitively open they are to incoming stimuli, how emotionally activated or stressed they are, and how well aligned they are with the natural day–night cycle. These three dimensions determine how the patient perceives the room. The room sends the patient light and sound. The patient can respond in only a few, highly constrained ways because the model represents an intubated individual with severely limited mobility. The patient’s hidden state is a triple:

$$s_{P}=\left(\gamma_{\mathrm{cog}},\;\gamma_{\mathrm{emo}},\;\mathrm{circ}\right)\in \left\{0,1,2,3\right\}\times \left\{0,1,2,3\right\}\times \left\{0,1,2\right\},$$

yielding $N_{S}=48$ discrete states.

In this model, “precision” is used in its standard active inference sense: the precision of a distribution is the inverse of its variance, and it governs how strongly a given signal is weighted during belief updating. A high-precision likelihood makes observations sharply informative; a low-precision (flat) likelihood makes them nearly uninformative. We treat $\gamma_{cog}$ and $\gamma_{emo}$ as state variables that index the precision of, respectively, the patient’s sensory likelihood and the gain on threat-related signals. The link to neurobiology is that these precision parameters are known to be under neuromodulatory control so that physiological state translates into a precision level rather than into the content of the generative model.

The three dimensions represent:

- $\gamma_{cog}\in \left\{0,1,2,3\right\}$ (collapsed, low, normal, high): cognitive precision, i.e. the precision (inverse variance) of the patient’s sensory likelihood $A_{P}$. We model it as modulated by cholinergic thalamocortical gating—the acetylcholine-dependent control of how faithfully sensory signals are relayed from thalamus to cortex [35]—which provides a neurobiological substrate for sensory precision. When $\gamma_{cog}$ is low, $A_{P}$ flattens toward the uniform distribution, reducing the information content of observations and making belief updating less effective.
- $\gamma_{emo}\in \left\{0,1,2,3\right\}$ (minimal, moderate, elevated, spiked): emotional precision, i.e., the gain applied to threat- and arousal-related signals. We model it as modulated by the amygdala–prefrontal axis and by HPA-axis cortisol activation—the stress-response pathway that raises physiological arousal [38]—so that a more activated emotional state increases the weight given to threatening stimuli during inference.
- circ∈{0,1,2} (misaligned, partial, aligned): circadian alignment with the external light–dark cycle.

The patient observes 16 discrete stimuli from the room, formed by the Cartesian product of four light levels × four sound categories:

$$O_{P}=\left\{\mathrm{dark},\;\dim\_\mathrm{warm},\;\mathrm{bright}\_\mathrm{neutral},\;\mathrm{bright}\_\mathrm{blue}\right\}\times \left\{\mathrm{silence},\;\mathrm{nature},\;\mathrm{noise}\_\mathrm{incoherent},\;\mathrm{alarm}\right\}.$$

The patient selects among three micro-actions reflecting the constrained motor repertoire of an intubated individual: $A_{P}=\left\{\mathrm{passive},\;\mathrm{orient},\;\mathrm{startle}\right\}$. The startle action is also triggered stochastically with phenotype-dependent probability, modeling involuntary autonomic responses.

#### 3.3.2. Patient’s Likelihood Matrix

In the patient agent, the likelihood matrix $A_{P}$ links the patient’s internal hidden state to the sensory stimuli received from the room. The critical feature of this matrix is that its quality depends directly on the level of cognitive precision: when the cognitive dimension is low, $A_{P}$ flattens toward a uniform distribution, meaning that incoming stimuli become uninformative. In practical terms, the patient still sees and hears something but what they see and hear does less and less work in helping them infer where they are and what is happening around them.

Therefore, $A_{P}\in {\left[0,1\right]}^{16\times 48}$ encodes $P(o_{P}|s_{P})$. Its critical property is *precision weighting*: column *s* of $A_{P}$ is a mixture,

(4) $$A_{P}\left[\cdot ,s\right]=\pi_{s}\cdot l_{a_{R}}+\left(1-\pi_{s}\right)\cdot u,$$

where the precision weight $\pi_{s}$ is obtained by indexing the vector (0.10,0.45,0.80,1.00) at the cognitive-precision level $\gamma_{cog}\left(s\right)\in \left\{0,1,2,3\right\}$ of state *s*; that is, $\pi_{s}=0.10$ when $\gamma_{cog}\left(s\right)=0$, rising to $\pi_{s}=1.00$ when $\gamma_{cog}\left(s\right)=3$; $l_{a_{R}}$ is the probability vector (the pmf) over $O_{P}$ consistent with the current room action, and *u* is the uniform probability vector over $O_{P}$. At $\gamma_{\mathrm{cog}}=0$, $A_{P}\left[\cdot ,s\right]\approx u$: observations carry minimal information and belief updating is severely impaired.

#### 3.3.3. Patients’ Transition Matrix $B_{P}$ and Preference Vector $C_{P}$

In the patient agent, the transition matrix $B_{P}$ describes how the patient’s hidden state may change following the patient’s own micro-actions—remaining passive, orienting, or startling. It is therefore a table that encodes the following question: given my current internal state and the action I just took, what state am I likely to be in at the next cycle? The preference vector *C* does not describe what happens but what the agent would like to happen.

$B_{P}\in {\left[0,1\right]}^{48\times 48\times 3}$ encodes state transitions under patient micro-actions, scaled by phenotype multipliers (see Table 1). $C_{P}\in R^{16}$ encodes log-preferences favouring dim_warm light (+2) and nature sound (+2), and penalizing alarm (−3) and bright_blue (−2).

#### 3.3.4. The ICU Room

Throughout this paper, the “room agent” is a modeling construct, not a claim that a physical room holds beliefs. In the active inference framework, an agent is defined structurally—by a Markov blanket across which sensing and action occur, together with the inference that links them—and the framework is agnostic about the physical substrate that implements that inference. The “room agent” should therefore be read as the environment-side sensing–inference–actuation loop: the physiological sensors that observe the patient, the generative model that maintains a belief about the patient’s hidden state, and the actuators that set light and sound. Crucially, the inferential component need not be automated. In current intensive care it is the clinical team that observes the patient’s physiological signals, forms and updates a belief about the patient’s state, and adjusts the room configuration accordingly; the reading in which the medical team entertains the beliefs and the room is the means through which they act is thus a specific instantiation of the agent defined here, not an alternative to it. The same loop could equally be instantiated by an automated monitoring system as described in Section 3.5.6. What the model requires is only that some inferential process on the environment side maintains and updates a model of the patient; whether that process is a clinician, an algorithm, or both, or a future automated room is an empirical and engineering question, not a commitment of the model.

Now, the room agent does two things simultaneously. First, at every cycle it attempts to infer the patient’s current internal state. Second, over time it also attempts to learn what kind of patient it is dealing with—specifically, whether this is someone who responds well to certain environmental interventions or someone who reacts more strongly and stressfully to incoming stimuli. This is the hierarchical dimension of the model: a fast estimate of the current state running in parallel with a slower learning process that builds a profile of the individual patient. To do this, the room observes the patient’s physiological signals and then selects a combination of light and sound. The underlying logic is straightforward: the room tries different configurations, observes how the patient responds, progressively learns what works for this particular individual, and in doing so constructs an increasingly personalized generative model.

The room implements a two-level generative model, which constitutes the architectural innovation of this paper. At Level 1 (fast timescale), the room infers the patient’s current hidden state $Q_{R}\left(s_{t}\right)$ every cycle. At Level 2 (slow timescale), the room simultaneously infers the patient’s latent individual parameters θ, building a progressively personalized generative model.

The room’s Level-1 hidden state is the same 48-dimensional space as the patient’s hidden state: the room maintains a belief $Q_{R}\left(s\right)$ about the patient’s current $\left(\gamma_{\mathrm{cog}},\gamma_{\mathrm{emo}},\mathrm{circ}\right)$.

The room also infers $\theta =\left(\theta_{cog},\theta_{emo}\right)\in \left[0.1,\;1.0\right]\times \left[0.1,\;1.0\right]$, where each component ranges over the continuous interval [0.1,1.0]. $\theta_{\mathrm{cog}}$ is the patient’s *cognitive plasticity* (how effectively room interventions improve $\gamma_{\mathrm{cog}}$) and $\theta_{\mathrm{emo}}$ is *emotional reactivity* (how strongly threatening stimuli elevate $\gamma_{\mathrm{emo}}$). These parameters are unknown at admission and must be inferred from physiological observations over time. For inference, each component of θ is discretized into 10 equally spaced values in [0.1,1.0], giving a 10×10 grid of $N_{\theta }=100$ points over the joint space.

#### 3.3.5. Room’s Observation and Action Space

The room receives 24 physiological signals from ICU sensors:

$$O_{R}=O_{\mathrm{HR}}\times O_{\mathrm{mov}}\times O_{\mathrm{EEG}}=\left\{0,1,2,3\right\}\times \left\{0,1,2\right\}\times \left\{0,1\right\},\;\left|O_{R}\right|=4\times 3\times 2=24.$$

The action space comprises $A_{R}=A_{\mathrm{light}}\times A_{\mathrm{sound}}$, yielding $|A_{R}|=16$ configurations.

#### 3.3.6. Room’s Transition Matrix

In the room agent, there is not a single transition matrix but an entire family of matrices $B_{R}\left(\theta \right)$, one for each possible combination of the latent patient parameters θ. This means the room does not simply encode the relationship between a light or sound configuration and its effect on the patient; it encodes the fact that this effect depends on what kind of patient is present. A patient with low cognitive plasticity will respond differently to warm dim light than a patient with high plasticity; a patient with high emotional reactivity will be more strongly destabilized by incoherent noise than one with low reactivity. The room therefore maintains many possible versions of the patient’s dynamics simultaneously, one for each θ combination, and uses an effective matrix $B_{R}^{\mathrm{eff}}$ that is a weighted average across all these possibilities, where the weights reflect everything the room has learned about this particular patient up to the current cycle.

Therefore, the room possesses a *family* of matrices ${\left\{B_{R}\left(\theta \right)\right\}}_{\theta \in \Theta }$, where each $B_{R}\left(\theta \right)\in {\left[0,1\right]}^{48\times 48\times 16}$ encodes how room actions shift patient states at a specific combination of plasticity and reactivity:

(5) $$\begin{array}{cc} \Delta \gamma_{\mathrm{cog}} & \propto \theta_{\mathrm{cog}}\cdot \left[\mathrm{light}\;\mathrm{action}\;\mathrm{benefit}\right], \end{array}$$

(6) $$\begin{array}{cc} \Delta \gamma_{\mathrm{emo}} & \propto \theta_{\mathrm{emo}}\cdot \left[\mathrm{sound}\;\mathrm{action}\;\mathrm{effect}\right]. \end{array}$$

The effective transition matrix used for inference is the θ-marginal:

(7) $$B_{R}^{\mathrm{eff}}=\sum_{\theta \in \Theta }Q_{R}\left(\theta \right)\cdot B_{R}\left(\theta \right).$$

#### 3.3.7. Room’s Likelihood and Preferences

In the room agent, the likelihood matrix $A_{R}$ does the mirror image of what $A_{P}$ does for the patient: it links the patient’s hidden state to the physiological signals that the room receives from its sensors. The room uses $A_{R}$ to ask itself: given that the patient is in a particular internal state, what physiological signals should I expect to observe? High heart rate and agitation point toward elevated emotional activation; altered EEG patterns point toward reduced cognitive precision. In this way, $A_{R}$ is the room’s model of how the patient’s internal condition expresses itself in measurable bodily signals.

$C_{R}$ does not encode preferences over light and sound configurations directly but over the patient’s hidden states: the room “prefers” the patient to have high cognitive precision, low emotional reactivity, and good circadian alignment. The room’s goal is therefore not to produce a particular sensory environment in the abstract but to bring the patient into a state that is neurobiologically stable and resistant to the onset of precision rigidity.

Therefore, $A_{R}\in {\left[0,1\right]}^{24\times 48}$ encodes $P(o_{R}|s_{R})$. $C_{R}\in R^{48}$ encodes log-preferences over patient states, assigning positive value to high $\gamma_{\mathrm{cog}}$, low $\gamma_{\mathrm{emo}}$, and aligned circadian.

#### 3.3.8. Clinical Phenotypes

The clinical phenotypes represent four distinct ICU patient profiles with different baseline vulnerabilities to delirium. In the model, each phenotype combines a specific cognitive-emotional starting point—encoded in the initial values of $\gamma_{\mathrm{cog}}$ and $\gamma_{\mathrm{emo}}$—with a different pattern of responsiveness to environmental stimulation, encoded in the true latent parameters $\left(\theta_{\mathrm{cog}},\theta_{\mathrm{emo}}\right)$ that the room must learn over time. This allows the simulation to test whether predictive synchronization breaks down differently across patient types: from younger and healthier patients, who start with high cognitive precision and respond well to environmental interventions, to more fragile or cognitively impaired ones, whose lower plasticity and higher emotional reactivity make them structurally more vulnerable to the onset of the precision rigidity cycle.

The four phenotypes used in the simulation are grounded in the literature review. Each phenotype’s initial precision values and true θ parameters reflect the neurobiological profile documented for that patient population.

### 3.4. Mathematical Structure of the Simulation

The model’s behavior emerges from the interaction of two inference processes operating at different timescales and in opposite directions. At the fast timescale, both agents update their beliefs every cycle. At the slow timescale, the room accumulates evidence about the patient’s latent parameters θ, progressively refining its generative model.

What connects these two timescales is the synchronization dynamic. When the two agents maintain accurate models of each other, belief updating proceeds normally. When synchronization degrades, a causal feedback loop is activated: the room’s failure to track the patient generates desynchronization pressure that progressively sharpens the patient’s prior beliefs, reducing the influence of incoming observations until the patient becomes unable to update its model of the environment.

#### 3.4.1. Variational Free Energy (VFE)

Active inference is grounded in the free energy principle (FEP). According to this principle, agents are able to persist in a chaotic environment because they are able to minimize a quantity called variational free energy [10]:

(8) $$F\left(o,Q\right)=\underset{\mathrm{inaccuracy}}{\underset{︸}{-E_{Q(s)}\left[\ln P(o|s)\right]}}+\underset{\mathrm{complexity}}{\underset{︸}{\mathrm{KL}\left[Q(s)\;∥\;D(s)\right]}}.$$

Here D(s) denotes the agent’s prior over hidden states (the prior *D* introduced in Section 3.3); Q(s) is the approximate posterior, and P(o∣s) the likelihood.

The inaccuracy term captures how surprised the agent is by the current observation, averaged over the states it believes it occupies: it is the negative log-likelihood −lnP(o∣s) taken in expectation under the posterior Q(s). If the states the agent considers likely would have generated the observation, inaccuracy is low; otherwise, it is high.

The complexity term captures how much the agent has had to revise its prior beliefs to accommodate the observation: a large revision incurs a high complexity cost. Minimizing VFE therefore means finding the posterior belief that explains the observation well without departing too far from the prior. This is the computational implementation of Bayesian inference under a variational approximation.

The clinical relevance of this decomposition becomes clear when precision rigidity sets in. When the patient’s prior beliefs become extremely peaked, the complexity term dominates: any observation that would require revising the prior incurs a prohibitive cost, and the posterior remains close to the prior regardless of what the observation says. The patient is not failing to receive sensory input. It is discounting that input because updating would be too costly given its current level of certainty. This is why delirium under the present model is not a sensory deficit but a failure of inference.

Now, the next step is to specify the concrete update rule through which this minimization is implemented at the level of state inference.

#### 3.4.2. Level-1 Belief Update (State Inference)

Both agents update state beliefs via variational message passing (VMP):

(9) $$Q^{*}\left(s\right)\propto A\left[\;o,\;s\;\right]\cdot Q_{\mathrm{prior}}\left(s\right),$$

followed by normalization over 16 VMP iterations per cycle.

In the discrete implementation, VMP reduces to a softmax update of log-likelihood plus log-prior. The 16 iterations therefore do not correspond to successive observations but to repeated within-cycle evaluations of the same posterior, used as a numerically convenient implementation of state inference.

#### 3.4.3. Predictive Prior Propagation

After action selection, each agent propagates its belief forward:

(10) $$Q_{\mathrm{prior}}^{\left(t+1\right)}\left(s^{\prime }\right)=\sum_{s}B\left[\;s^{\prime },\;s,\;a\;\right]\cdot Q^{*}\left(s\right).$$

For the room agent, *B* is replaced by $B_{R}^{\mathrm{eff}}$ from Equation (7).

This specifies how the posterior belief obtained at the current cycle is transformed into the prior for the next one: after state inference, each agent projects its updated belief forward through the action-conditioned transition model, thereby generating an anticipatory belief about the next hidden state.

#### 3.4.4. Level-2 Belief Update (θ Inference)

After each Level-1 update, the room updates its belief over latent patient parameters. The likelihood of each grid point θ is

(11) $$L^{\left(t\right)}\left(\theta \right)=B_{R}\left(\theta \right)\;\left[s_{t}^{\mathrm{MAP}},\;s_{t-1}^{\mathrm{MAP}},\;a_{R}^{\left(t\right)}\right].$$

The Bayesian update is applied in log-space with reliability weight $w_{t}\in \left[0,1\right]$:

(12) $$\ln Q_{R}^{\left(t\right)}\left(\theta \right)=w_{t}\cdot \ln L^{\left(t\right)}\left(\theta \right)+\ln Q_{R}^{\left(t-1\right)}\left(\theta \right)+\mathrm{const},$$

where $w_{t}=1-\min\;\left(H\left[Q_{R}^{\left(t\right)}\left(s\right)\right]/H\left[{\mathrm{Unif}}_{48}\right],\;0.9\right)$. Learning progress is tracked as

(13) $$\ell^{\left(t\right)}=1-\frac{H[Q_{R}^{\left(t\right)}\left(\theta \right)]}{H[{\mathrm{Unif}}_{N_{\theta }}]}\in \left[0,1\right].$$

The room starts with a uniform belief over all 100 points on the θ grid—it does not yet know anything about the specific patient. At each cycle, it observes how the patient responded to the action that was just executed and updates its estimate of which values of $\left(\theta_{\mathrm{cog}},\theta_{\mathrm{emo}}\right)$ make that response more or less likely. Over time, QR(θ) becomes more refined, and the effective matrix $B_{\mathrm{eff}}$ becomes increasingly personalized to that patient.

This mechanism has a direct consequence for the room’s behavior: in the early stages of the simulation, when *ℓ* is low and QR(θ) is still diffuse, the room is motivated to explore different configurations of light and sound—not because it already knows they are helpful but in order to gather information about the patient. As *ℓ* increases, the room gradually shifts to exploitation, using the personalized model to select the most effective actions. This transition is governed by the adaptive temperature $\tau^{\left(t\right)}$ described in the following section.

#### 3.4.5. Level-3 Action Selection

The room agent selects actions by minimizing expected free energy (EFE) [10,39], a quantity that simultaneously captures two distinct motivations for acting—bringing about preferred outcomes and reducing uncertainty about the patient’s current internal state:

(14) $$G\left(a_{R}\right)=\underset{\mathrm{pragmatic}\;\mathrm{term}}{\underset{︸}{-E_{Q_{R}\left(s^{\prime }\right)}\left[C_{R}\left(s^{\prime }\right)\right]}}-\underset{\mathrm{epistemic}\;\mathrm{term}}{\underset{︸}{I(o_{R};\;s_{R}|a_{R})}}$$

where $a_{R}\in A_{R}$ is the room’s chosen action (a joint light-sound configuration), $s^{\prime }\in S$ denotes the patient’s future hidden state, $Q_{R}\left(s^{\prime }\right)$ is the room’s predictive belief distribution over those future states obtained by propagating the current posterior through the effective transition matrix $B_{R}^{\mathrm{eff}}$ (Equation (10)), and $C_{R}\left(s^{\prime }\right)\in R$ is the room’s log-preference over patient hidden states, assigning positive value to states of high cognitive precision $\gamma_{\mathrm{cog}}$, low emotional reactivity $\gamma_{\mathrm{emo}}$, and circadian alignment circ.

The pragmatic term, $-E_{Q_{R}\left(s^{\prime }\right)}\left[C_{R}\left(s^{\prime }\right)\right]$, is the negative expected log-preference: minimizing *G* with respect to this term drives the room toward actions whose predicted consequences align with the room’s goal of maintaining the patient in a neurobiologically stable state. The epistemic term, $I(o_{R};\;s_{R}|a_{R})$, is the mutual information between the room’s future observations $o_{R}\in O_{R}$ and the patient’s hidden state $s_{R}$, conditioned on the chosen action. It decomposes as

(15) $$I\left(o_{R};\;s_{R}|a_{R}\right)=\underset{\mathrm{prior}\;\mathrm{uncertainty}}{\underset{︸}{H[Q_{R}\left(s^{\prime }\right)]}}-\underset{\mathrm{expected}\;\mathrm{posterior}\;\mathrm{uncertainty}}{\underset{︸}{E_{Q_{R}\left(o_{R}\right)}\left[H[Q_{R}\left(s^{\prime }|o_{R}\right)]\right]}}$$

where *H* denotes Shannon entropy, $Q_{R}\left(o_{R}\right)=\sum_{s^{\prime }}Q_{R}\left(s^{\prime }\right)A_{R}\left[o_{R},s^{\prime }\right]$ is the room’s predictive distribution over future observations, and $Q_{R}\left(s^{\prime }|o_{R}\right)$ is the room’s posterior belief over the patient’s hidden state after observing $o_{R}$, obtained via Bayes’ rule. The epistemic term thus quantifies the expected reduction in uncertainty about the patient’s state that would result from taking action $a_{R}$ and observing the patient’s physiological response. Minimizing *G* with respect to this term drives the room to prefer actions that are maximally informative about the patient’s current internal state, independently of whether those actions are immediately therapeutic.

The action is sampled from a softmax policy with adaptive temperature:

(16) $$\pi \left(a_{R}\right)\propto \exp\left(-G\left(a_{R}\right)\;/\;\tau^{\left(t\right)}\right),\;\tau^{\left(t\right)}=\tau_{0}\cdot \left(2-\ell^{\left(t\right)}\right)$$

where $\tau_{0}>0$ is the baseline temperature and $\ell^{\left(t\right)}\in \left[0,1\right]$ is the learning progress index defined in Section 3.4.4, measuring how much the room’s belief $Q_{R}\left(\theta \right)$ over the patient’s latent parameters has concentrated away from the uniform prior. The adaptive temperature $\tau^{\left(t\right)}$ implements a principled exploration–exploitation tradeoff. In the early cycles of a patient’s ICU stay, when $\ell^{\left(t\right)}$ is close to zero and the room’s belief over θ remains diffuse, $\tau^{\left(t\right)}$ is close to $2\tau_{0}$ and the softmax distribution is relatively flat: the room is motivated to try varied light and sound configurations not because it knows they will benefit the patient but because the physiological responses they elicit will reduce uncertainty about the patient’s true $\left(\theta_{\mathrm{cog}},\theta_{\mathrm{emo}}\right)$. As $\ell^{\left(t\right)}$ grows over successive cycles and $Q_{R}\left(\theta \right)$ sharpens around the patient’s true parameter values, $\tau^{\left(t\right)}$ decreases toward $\tau_{0}$ and the room’s policy concentrates on the actions that its now-personalized generative model identifies as most likely to maintain the patient in a state of high cognitive precision and low emotional reactivity.

This transition from uncertainty-driven exploration to model-based exploitation is the mechanism through which the Level-3 hierarchical architecture implements personalized environmental management: the room does not apply a fixed sensory protocol but continuously refines its action policy as its model of the individual patient improves.

#### 3.4.6. Delirium Criterion

This section formally defines when delirium is declared in the model.

The forward direction of the model runs from low $\gamma_{\mathrm{cog}}$ to de-synchronization: when $\gamma_{\mathrm{cog}}$ is low, $A_{P}$ carries less information, belief updating is impaired, and the patient’s state becomes harder for the room to predict. The reverse direction, specified below, runs from de-synchronization back to precision rigidity through prior sharpening. Delirium is the clinical endpoint of this self-reinforcing cycle, declared when both directions of prediction have failed simultaneously and persistently.

**Definition** **2** (Delirium onset)**.** Delirium is declared at cycle $t^{*}$ when the following two conditions hold simultaneously for $N_{\mathrm{sus}}=4$ consecutive cycles:

(17) $$\begin{array}{cc} \left(i\right)\; & S_{R\to P}^{\left(t\right)}>\theta_{R}=3.0\;\mathrm{nats}\;(\mathrm{room}\;\mathrm{cannot}\;\mathrm{predict}\;\mathrm{patient}), \end{array}$$

(18) $$\begin{array}{cc} \left(\mathrm{ii}\right)\; & S_{P\to R}^{\left(t\right)}>\theta_{P}=3.0\;\mathrm{nats}\;(\mathrm{patient}\;\mathrm{cannot}\;\mathrm{predict}\;\mathrm{room}). \end{array}$$

#### 3.4.7. Desynchronization Pressure

Precision rigidity is not a brain that stops receiving signals. It is a brain that receives signals but holds its prior beliefs with such high confidence that no observation can update them. The causal feedback therefore does not degrade the patient’s generative model ($A_{P}$, $B_{P}$ remain unchanged). Instead, it progressively sharpens the patient’s prior belief distribution, making it increasingly peaked around the current MAP state.

This section specifies the reverse causal direction of the model: persistent de-synchronization causes precision rigidity in the patient. While Section 3.4.6 established the criterion for declaring delirium, the mechanism described here explains how the patient arrives at that state—not through a breakdown of its generative model but through a progressive sharpening of its prior beliefs that renders incoming observations unable to update them.

At each cycle *t*, pressure $\rho^{\left(t\right)}\in \left[0,1\right]$ is computed from the recent history of $S_{R\to P}$:

(19) $$\rho^{\left(t\right)}=\frac{1}{W}\sum_{k=t-W}^{t-1}\min\;\left(\frac{\max\;\left(S_{R\to P}^{\left(k\right)}-\theta_{R},\;0\right)}{\Delta_{\max}},\;1\right),$$

where W=10, $\theta_{R}=3.0$ nats, $\Delta_{\max}=3.0$ nats.

The pressure $\rho^{\left(t\right)}$ is applied to the patient’s current belief distribution via exponentiation:

(20) $$Q_{P}^{\mathrm{sharp}}\left(s\right)\propto {\left[Q_{P}^{\left(t\right)}\left(s\right)\right]}^{\;1+\rho^{\left(t\right)}\cdot \alpha },$$

with α=0.05. Raising a distribution to a power >1 concentrates probability mass on high-probability states, making the prior progressively more peaked. The sharpened distribution $Q_{P}^{\mathrm{sharp}}$ replaces $Q_{P}^{\left(t\right)}$ and propagates forward as the prior for the next cycle’s belief update.

As $\rho^{\left(t\right)}\to 1$ over successive cycles, the exponent grows and the prior approaches a delta function on the MAP state: $Q_{P}^{\mathrm{sharp}}\approx \delta_{s^{*}}$. At this point, the VMP update (Equation (9)) yields:

$$Q^{*}\left(s\right)\propto A_{P}\left[\;o,\;s\;\right]\cdot Q_{P}^{\mathrm{sharp}}\left(s\right)\approx A_{P}\left[\;o,\;s^{*}\;\right]\cdot \delta_{s^{*}}\propto \delta_{s^{*}},$$

meaning the posterior is insensitive to the observation regardless of its content. This is precision rigidity in the active inference sense: the patient is not confused—the patient is locked.

The patient’s generative model ($A_{P}$, $B_{P}$, $C_{P}$) is unchanged. The patient’s model of how the world works remains intact. Only its confidence in its current state becomes pathologically elevated. This is consistent with clinical presentations of hypoactive delirium, in which the patient is not agitated or hallucinating (which would indicate a broken generative model) but instead appears withdrawn and unresponsive—locked into an internal state that environmental input can no longer shift.

The full causal chain is

(21) $$S_{R\to P}^{\left(t\right)}↑\;\overset{\;\rho^{\left(t\right)}\;}{\to }Q_{P}^{\mathrm{sharp}}\to \delta_{s^{*}}\;\overset{\;\mathrm{precision}\;\mathrm{rigidity}\;}{\to }Q^{*}\left(s\right)\approx Q_{\mathrm{prior}}\left(s\right)\;\overset{\;\mathrm{joint}\;\mathrm{criterion}\;}{\to }\mathrm{delirium}.$$

### 3.5. Results

**Figure 1:**
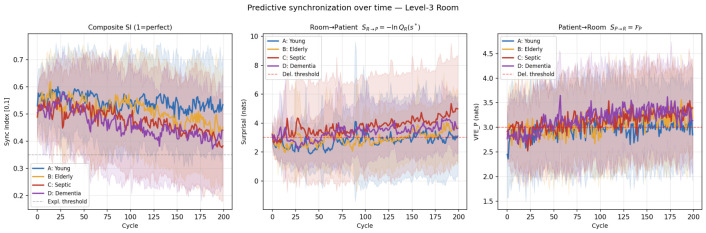
Predictive synchronization dynamics over 200 decision cycles (approximately 3 h of ICU time) across four clinical phenotypes (n=30 patients per phenotype, prior sharpening active). (**Left**): composite synchronization index SI∈[0,1], where 1 indicates perfect bidirectional prediction. Phenotype A (young healthy, blue) maintains consistently higher SI throughout the simulation, while phenotypes C (septic, red) and D (dementia, purple) decline to lower stable levels. (**Center**): room surprisal at the patient’s true state, $S_{R\to P}=-\ln Q_{R}\left(s^{*}\right)$. After a transient in the first 20–30 cycles, the four phenotypes diverge: phenotype A stabilizes below the delirium threshold (red dashed line, 3.0 nats) on average, while phenotypes C and D have mean trajectories that lie above it for much of the simulation. Because the ±1 SD bands overlap substantially across phenotypes, this should be read as a difference in means rather than a separation at the individual-patient level; the robustness of the ordering (in particular A < D) is assessed across seeds in Section 3.8.1. (**Right**): patient surprisal at room observations, $S_{P\to R}=-\ln P\left(o_{R}|Q_{P}\right)$ (true symmetric metric, Equation (2)). Phenotypic separation is substantially smaller than in $S_{R\to P}$ (range 0.21 versus 1.03 nats), consistent with the finding that the room’s predictive failure is the primary driver of de-synchronization. All curves rise toward and across the delirium threshold in the second half of the simulation, reflecting the accumulation of prior sharpening pressure in vulnerable phenotypes. Shaded areas represent ±1 SD across patients.

A cohort of 120 virtual patients (30 per phenotype) was simulated for 200 decision cycles (approximately 3 h of ICU time) using a fixed random seed. All code is available at https://github.com/DesignAInf/DELIRIUM-AND-SYNCHRONIZATION.git (accessed on 15 June 2026). These synchronization dynamics across the four phenotypes are shown in Figure 1.

Before presenting the results, it is important to clarify what the model can and cannot demonstrate. The quantitative outputs should not be interpreted as empirical predictions. What the simulation demonstrates is whether the proposed causal architecture produces qualitative patterns that are internally consistent with the hypothesis and coherent with established clinical knowledge.

#### 3.5.1. Statistical Approach

**Figure 2:**
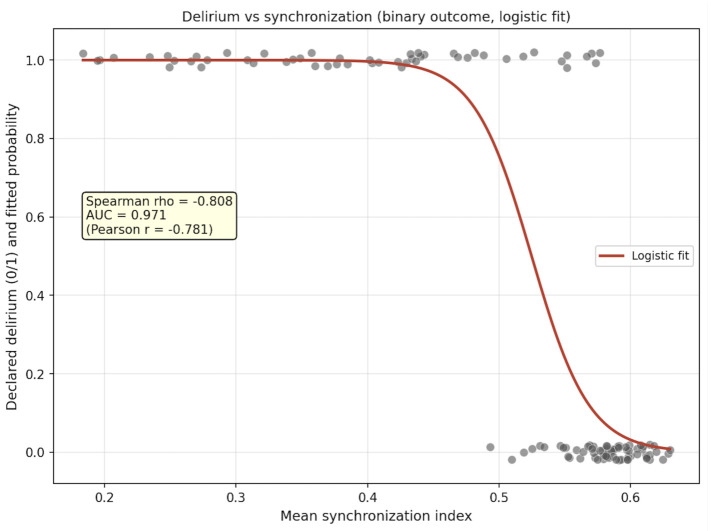
Relationship between the mean synchronization index and declared delirium across the 120-patient cohort (full model, 200 cycles). Each point is one patient; the outcome is the binary declared-delirium flag (Definition 2), shown with small vertical jitter for visibility. The solid curve is the fitted logistic regression P(delirium)∼meanSI, which represents the thresholded, saturating relationship far better than a linear fit. The mean synchronization index discriminates the two outcome classes at AUC=0.97; the monotonic association is ρ=−0.81 (Pearson r=−0.78 on the same binary outcome, shown for comparison). Lower synchronization is strongly associated with higher delirium probability, consistent with the predictive-synchronization hypothesis.

The relationship between the mean synchronization index and delirium is not linear. It is a thresholded, saturating process: patients who enter the precision-rigidity regime accumulate at the maximal outcome, producing the bimodal distribution visible in Figure 2. The Pearson correlation, which quantifies *linear* association and is sensitive to a saturated cluster of this kind, is therefore not an appropriate summary of the relationship. We instead model the genuine binary outcome—whether sustained joint-threshold delirium was declared (Definition 2)—as a function of the mean synchronization index via logistic regression, and we report Spearman’s ρ as a distribution-free measure of monotonic association. Modeling the declared-delirium outcome also removes a structural dependence between the two axes since the continuous desynchronization proxy used previously and the synchronization index are both derived from the same directional surprisals. Pearson values are retained only where useful for continuity of comparison.

#### 3.5.2. Result 1: Phenotypic Gradient in Delirium Rate

The phenotypic gradient in delirium rate is preserved across all four phenotypes: young healthy patients develop declared delirium in 27% of cases, while septic and dementia patients reach 53%. The gradient is monotonic in synchronization index (SI: 0.54 → 0.46) and in room surprisal ($S_{R\to P}$: 2.75 → 3.78 nats), confirming that more vulnerable phenotypes are associated with lower mutual predictive capacity between patient and environment.

Logistic regression of declared delirium on the mean synchronization index across all 120 patients yields a coefficient $\beta_{1}=-45.6$ (95% CI [−64.5,−26.6]), with the mean synchronization index discriminating delirious from non-delirious patients at AUC=0.97. The Spearman rank correlation between mean synchronization index and declared delirium is ρ=−0.81 (Pearson r=−0.78 on the same binary outcome, reported for comparison). All three confirm the hypothesized direction—lower synchronization is strongly associated with higher delirium risk—without assuming a linear relationship. We note that, once the appropriate binary outcome is used, the Pearson and Spearman coefficients are close, indicating that the earlier discrepancy was an artifact of summarizing a thresholded relationship with a linear statistic rather than a genuine non-monotonicity.

An important caveat applies: the direction of the gradient was scaffolded by the phenotypic parametrization, which reflects established clinical risk profiles (Table 1). What the simulation contributes is the specific magnitude of the gradient and the stochastic pattern of which individual patients within each phenotype develop declared delirium, both of which depend on the coupling dynamics rather than on initial conditions alone. The causal contribution of each architectural component is assessed systematically in Section 3.6.

#### 3.5.3. Result 2: Room Inference Drives the Synchronization Signal

The key test of the architecture is whether an inferring room is required to reproduce the synchronization–delirium relationship at all.

Using the distribution-free statistic on the declared-delirium outcome, the association collapses when the room does not infer: in the random-room baseline (M0) the Spearman correlation is ρ=−0.43 and delirium rates saturate at 89–94% across phenotypes, whereas every variant in which the room maintains a generative model of the patient yields a substantially stronger and more stable association (ρ between −0.78 and −0.84; see Table 2). The precision-rigidity (prior-sharpening) mechanism does not further increase the strength of this correlation—its effect is of a different kind. It does not modify the patient’s generative model ($A_{P}$ and $B_{P}$ are unchanged), only the patient’s confidence in its current state; as de-synchronization pressure accumulates, the patient becomes progressively more certain and less able to update from incoming observations. Its contribution, established in the ablation study (Section 3.6), is to stabilize the phenotypic gradient and amplify the separation between vulnerable phenotypes rather than to raise the overall correlation. This contribution should therefore be read as evidence of internal coherence rather than as empirical validation of the mechanism.

#### 3.5.4. Result 3: Dissociability of the Two Synchronization Directions

The delirium criterion requires the simultaneous failure of both prediction directions (conditions (17) and (18)). This is a theoretical choice by definition. What emerges from the simulation is that the two conditions are dissociable in practice: there are substantial periods in which $S_{R\to P}$ exceeds threshold while $S_{P\to R}$ does not. Across all declared delirium cases, $S_{R\to P}$ exceeds the threshold before $S_{P\to R}$ in the majority of patients, consistent with the causal sequence specified in Equation (21): the room loses track of the patient first, then prior sharpening accumulates, then the patient loses the ability to predict the room.

This dissociability can have a clinical interpretation. A patient can be in a state where the room’s model has severely diverged from their internal state—$S_{R\to P}$ high—while the patient is still generating physiologically predictable signals—$S_{P\to R}$ below threshold. This corresponds to the clinical presentation of hypoactive delirium: a patient who appears calm and generates few unexpected signals but whose internal model has already begun locking into a state that environmental input can no longer shift. The model predicts that monitoring $S_{R\to P}$ should detect this state earlier than monitoring the patient’s behavioral output alone.

#### 3.5.5. Result 4: $S_{R\to P}$ Is the Primary Informative Metric

Across phenotypes, the mean of $S_{R\to P}$ ranges from 2.75 nats (phenotype A) to 3.78 nats (phenotype C), a spread of 1.03 nats, whereas the mean of $S_{P\to R}$ ranges from 2.97 to 3.18 nats, a spread of only 0.21 nats (Table 3). In terms of mean separation, the room surprisal therefore differentiates phenotypes roughly five times more than the patient surprisal, even with the corrected commensurable metric. This separation is in the means: the within-phenotype standard deviation of $S_{R\to P}$ is itself of order 1–3 nats (Table 3), so the phenotypes overlap substantially at the individual-patient level. The claim we make is correspondingly ordinal—the room surprisal carries more phenotype-discriminating signal than the patient surprisal—and its robustness rests on the cross-seed analysis, not on a clean separation of individual trajectories. This asymmetry is genuine: as established in Section 3.1, both metrics are now negative log-probabilities of an observation under a belief distribution, so the difference in discriminative power reflects a structural property of the coupling, not a measurement artifact.

The clinical implication is that environment-side monitoring—systems that continuously update a generative model of the patient from physiological sensor arrays—may detect impending delirium earlier and more reliably than patient-side behavioral monitoring alone. The specific form of $S_{R\to P}$ that would be estimable from clinical data is discussed in Section 3.5.7.

#### 3.5.6. Result 5: θ Learning Progress

Mean θ learning progress reaches ℓ=0.07–0.10 over 200 cycles. The room has begun constructing a patient-specific generative model, with lower learning in phenotype D (dementia, ℓ=0.07) reflecting the greater unpredictability of these patients’ physiological trajectories, which reduces the reliability weight $w_{t}$ in Equation (12) and slows θ convergence.

Full convergence of $Q_{R}\left(\theta \right)$ requires substantially longer simulations, corresponding to a multi-day ICU stay. As $Q_{R}\left(\theta \right)$ sharpens, $B_{R}^{\mathrm{eff}}$ becomes increasingly patient specific. This should reduce the room’s baseline surprisal $S_{R\to P}$ for patients who have not yet developed delirium, thereby reducing the desynchronization pressure applied through the prior sharpening mechanism. A room that has learned the patient’s parameters is therefore less likely to generate the persistent de-synchronization that initiates the precision rigidity cycle. This constitutes a theoretically motivated prevention mechanism: personalized environmental management reduces the probability of triggering the causal chain leading to delirium. This prediction is not yet verified by simulation and is left for future work.

#### 3.5.7. Bridge to Empirical Implementation

A fundamental challenge for translating the model’s predictions to clinical practice is that $S_{R\to P}=-\ln Q_{R}\left(s^{*}\right)$ is computed with respect to the patient’s true hidden state $s^{*}$, which is accessible in simulation but not directly observable in clinical settings. For this metric to serve as an early-warning signal in real ICUs, it must be estimated from observable physiological signals.

The key insight is that $S_{R\to P}$ measures the entropy of the room’s belief distribution $Q_{R}\left(s_{t}\right)$ over the patient’s internal state. When $Q_{R}\left(s_{t}\right)$ is concentrated, the room is tracking the patient well; when it is diffuse, the room has lost track. An empirical analog of $S_{R\to P}$ can therefore be approximated as the entropy of a continuously updated Bayesian model of the patient, trained on observable physiological signals.

Each dimension of the patient’s hidden state has known physiological correlates measurable continuously in the ICU:

- $\gamma_{\mathrm{cog}}$ correlates with EEG spectral characteristics, particularly the ratio of slow-wave to alpha/beta power and the suppression of thalamocortical spindles [35].
- $\gamma_{\mathrm{emo}}$ correlates with heart rate variability, galvanic skin response, and cortisol markers of HPA activation [38].
- circ correlates with the melatonin-driven light–dark rhythm, estimable from wrist actigraphy or continuous skin temperature [40].

A room agent trained offline on ICU physiological recordings paired with environmental logs would maintain a running belief $Q_{R}\left(s_{t}\right)$ updated at each observation. The empirical analog of $S_{R\to P}$ would then be the entropy of this distribution: low entropy indicates accurate patient tracking; rising entropy signals emerging de-synchronization before clinical delirium onset.

The model generates a concrete falsifiable prediction for prospective clinical testing. In real ICU data, a continuous environment-side monitoring system—one that maintains a running Bayesian model of the patient updated from physiological sensor arrays (EEG, heart rate variability, and actigraphy) and generates an alarm when the entropy of that model exceeds a threshold, signaling that the room has lost track of the patient—should trigger before the CAM-ICU (Confusion Assessment Method for the ICU), the standard bedside tool for detecting delirium, turns positive. In other words, the model predicts that the environment’s predictive failure becomes detectable before the patient’s behavioral symptoms become clinically visible. This temporal precedence is the most direct empirical test of the predictive synchronization hypothesis, and it is falsifiable: a prospective ICU study with simultaneous continuous physiological monitoring and standard clinical delirium assessment could either confirm or refute it.

### 3.6. Model Comparison: Ablation Study

The ablation study addresses three methodological requirements that the main simulation alone cannot satisfy. First, it rules out the possibility that the results are entirely determined by the phenotypic initial conditions rather than by the coupling dynamics: without this test, the phenotypic gradient in delirium rate could be dismissed as a trivial consequence of the parametrization in Table 1 rather than an emergent property of the two-agent architecture. Second, it isolates the independent contribution of each architectural component—room inference, θ learning, and prior sharpening—by removing them one at a time and observing the consequences for the three qualitative criteria: gradient direction, correlation magnitude, and cross-seed consistency. Third, it meets the methodological standard expected in computational modeling: a paper proposing a multi-component architecture without testing the contribution of each component would be vulnerable to the objection that the full model’s performance reflects redundancy rather than necessity.

We compare the full model against three progressively impoverished variants:

- **M0** (Random room): The room selects actions uniformly at random and performs no inference. This is the single-agent baseline in which only the patient minimizes free energy.
- **M1** (No θ learning): The room performs state inference but does not update $Q_{R}\left(\theta \right)$. The effective transition matrix $B_{R}^{\mathrm{eff}}$ remains fixed at the prior mean, never personalizing to the patient.
- **M2** (No causal feedback): The room performs full inference including θ learning but the desynchronization pressure $\rho^{\left(t\right)}$ is not applied to the patient’s prior. De-synchronization does not induce precision rigidity.
- **M3** (Full model): All components are active—θ inference, prior sharpening causal feedback, and bidirectional coupling. Persistent de-synchronization progressively concentrates the patient’s prior belief distribution, implementing precision rigidity in the active inference sense: the patient is not confused by noise but locked into a state that observations can no longer update.

Each variant was run with n=12 patients per phenotype, 150 decision cycles, and 3 independent random seeds ({1,7,42}). Table 2 reports mean delirium rates per phenotype, mean and SD of the rank correlation ρ(SI,declareddelirium), and the fraction of seeds in which the phenotypic gradient A<B,C,D is preserved.

Three findings emerge from the comparison. First, room inference is necessary. When the room acts randomly (M0), delirium rates saturate at 86–94% across all phenotypes and the synchronization–delirium association is weak and unstable (ρ=−0.43±0.10); the phenotypic gradient is preserved in only one of three seeds. Every variant in which the room maintains a generative model of the patient (M1–M3) produces both a graded phenotypic response and a substantially stronger, more stable association (ρ between −0.78 and −0.84). This confirms that an inferring room agent is required to reproduce phenotypic differentiation: without it, de-synchronization is driven purely by patient vulnerability, producing ceiling effects rather than a graded response.

Second, θ learning and the prior-sharpening feedback do not act primarily by strengthening the correlation—across M1, M2 and M3 the Spearman correlation is comparable. Their contribution is of a different kind: they reduce ceiling effects in the most vulnerable phenotypes and shape the *gradient* rather than the overall association. Removing θ learning (M1) leaves the room using a generic, non-personalized prior that is systematically wrong for vulnerable phenotypes, inflating delirium rates in phenotypes B–D relative to the full model; adding personalization (M3) lowers these rates and sharpens the separation between phenotype A and the vulnerable phenotypes.

Third, the prior-sharpening feedback amplifies rather than creates the effect. M2 (no feedback) and M3 (full) produce similar aggregate correlations but the feedback loop accelerates locking in patients already near threshold, which is the mechanism by which it stabilizes the gradient. Consistent with our framing throughout, this contribution is evidence of internal coherence—the model behaves as the hypothesis predicts when the mechanism is active—rather than an independent empirical validation of the mechanism.

A caveat is warranted on the Grad column: at n=12 per phenotype and three seeds, the gradient-preservation fraction is a coarse, high-variance indicator, and the intermediate ordering of phenotypes B, C and D fluctuates across seeds. The robust, replicable claim is the one supported by the main cohort (Section 3.5.1) and the seed-robustness analysis (Section 3.8.1): phenotype A develops less delirium than phenotype D across configurations. Larger cohorts would be required to estimate the intermediate ordering with confidence.

### 3.7. Temporal Signature: Does $S_{R\to P}$ Precede $S_{P\to R}$?

A model that proposes a directed causal sequence (Equation (21)) should produce a temporal ordering consistent with it: the room should lose predictive accuracy before the patient does. This ordering is already visible in Result 3 (Section 3.5.3): across declared-delirium cases, $S_{R\to P}$ crosses the delirium threshold before $S_{P\to R}$ in the majority of patients. Defining the per-patient lead time as $\Delta t=t^{*}\left(S_{P\to R}>\theta_{P}\right)-t^{*}\left(S_{R\to P}>\theta_{R}\right)$, where $t^{*}\left(x>\theta \right)$ is the first cycle at which metric *x* exceeds θ=3.0 nats, this lead time is positive in the large majority of cases, and no patient develops $S_{P\to R}>3.0$ before $S_{R\to P}>3.0$ in any variant with room inference.

Two caveats are important. First, this ordering is largely a structural property of the two surprisal measures rather than a distinctive signature of the full model: $S_{R\to P}$ responds directly to divergence between the room’s model and the patient’s true state, whereas $S_{P\to R}$ can rise only after prior sharpening has accumulated sufficiently to impair the patient’s belief updating, so the room-side signal is expected to move first by construction. Second, as discussed in Section 3.1, $S_{R\to P}$ is anchored to the patient’s true hidden state $s^{*}$, which is available in simulation but not directly observable clinically. The temporal precedence should therefore be read as an internal coherence check on the causal architecture, not as an independent empirical result.

Its clinical translation, however, is a genuinely falsifiable prediction. If an environment-side signal computed from observable physiology (the empirical analog of $S_{R\to P}$ discussed in Section 3.5.6) rises before patient-side behavioral symptoms, then a continuous environment-side monitor should generate an alarm before standard bedside screening (CAM-ICU) turns positive. Testing this temporal precedence in a prospective ICU study with simultaneous continuous physiological monitoring and standard clinical delirium assessment is the most direct empirical test of the predictive-synchronization hypothesis.

### 3.8. Robustness and Sensitivity of the Simulation Results

The quantitative results reported in Section 3.5 were obtained with a fixed random seed and a specific set of causal feedback parameters. This section assesses the stability of those results across independent replications and across parameter variations, and discusses what the analysis does and does not establish.

#### 3.8.1. Robustness Across Random Seeds

To assess whether the reported results depend on the specific random seed, we replicated the 200-cycle simulation (n=20 patients per phenotype, prior sharpening active) across five independent seeds {1,7,13,42,99}. Table 4 reports the mean, standard deviation, minimum, and maximum delirium rate per phenotype, together with the Spearman correlation ρ(SI,declareddelirium) and the overall delirium rate across all five replications. As in Section 3.5 and Section 3.6, the outcome is the genuine binary declared-delirium flag (Definition 2) and the association is summarized with the distribution-free Spearman ρ rather than Pearson’s *r*.

Three findings are stable across all five replications. First, phenotype A always has the lowest mean delirium rate and phenotype D always has the highest; the phenotypic gradient A<D is preserved in every seed. The intermediate ordering of B and C is not resolved at this sample size—their mean rates are in fact equal (51%)—reflecting the stochastic nature of the coupling dynamics rather than a stable difference. Second, the Spearman correlation between synchronization and declared delirium is strongly negative in every replication, ranging from −0.852 to −0.763 with a mean of −0.797 (SD =0.031); this is consistent with both the main cohort (ρ=−0.81, Section 3.5.1) and the full model in the ablation study (ρ=−0.80, Section 3.6). Third, the overall delirium rate is stable (range 46–54%, SD =2.8%).

The main source of variability is within-phenotype variance, particularly for phenotypes C and D. With n=20, each percentage point corresponds to 0.2 patients, so reported rates should be interpreted as ordinal indicators of relative risk rather than precise probability estimates. Larger cohorts (n≥100 per phenotype) would be required to narrow confidence intervals to clinically meaningful precision and to resolve the intermediate B/C ordering.

#### 3.8.2. Sensitivity of Causal Feedback Parameters

The prior sharpening mechanism is governed by three parameters: the desynchronization history window *W*, the sharpening strength α, and the desynchronization threshold $\theta_{R}$. These were set from theoretical considerations (W=10, α=0.05, $\theta_{R}=3.0$ nats) rather than estimated from data, and they are therefore not empirically identifiable from the present simulations.

There is a structural reason to expect the model’s qualitative conclusions to be insensitive to the precise values of these parameters within a broad range. At the simulation horizons used here, the outcome is driven by whether desynchronization pressure is activated at all—that is, by whether the room persistently fails to track the patient—rather than by the exact magnitude or time window of the sharpening. The direction of the phenotypic gradient and the sign of the synchronization–delirium association follow from the structure of the coupling, not from the specific feedback constants. At longer horizons, differences in α and $\theta_{R}$ would be expected to produce quantitative differences in delirium rates—stronger sharpening accelerating the locking process in vulnerable phenotypes—while leaving the direction of the gradient intact. A systematic one-at-a-time sensitivity sweep over *W*, α, and $\theta_{R}$, and the calibration of these parameters against physiological data, are left as explicit next steps.

#### 3.8.3. Model-Form Robustness (Structural Mismatch)

The robustness analyses above vary random seeds and feedback parameters but hold the form of both generative models fixed. A distinct and important question is whether the conclusions survive changes in model complexity: the patient and room models used here are deliberately simplified (a 48-state patient, a 10×10 parameter grid), and a more or less granular model might behave differently. We did not vary model form in the present study, and we flag this as the principal untested robustness dimension. Two considerations bound our expectation. On one hand, the conclusions we rely on are ordinal (the gradient A<D, the sign of the synchronization–delirium association) and follow from the structure of the coupling—an inferring room that loses track of a vulnerable patient—rather than from the resolution of the state space, which leads us to expect them to be qualitatively preserved under refinement. On the other hand, this expectation is itself a hypothesis: richer models could introduce dynamics (for example, additional hidden factors or non-stationary parameters) that change which phenotypes lock and when. A systematic study that constructs patient and room models at several levels of complexity and tests whether the gradient and association are preserved across them is therefore a necessary next step, alongside the calibration against physiological data described in Section 3.5.6.

#### 3.8.4. What the Analysis Establishes and What It Does Not

Taken together, the robustness analysis supports three claims with different levels of confidence.

The claim supported most strongly is the direction of the gradient: phenotype A always develops less delirium than phenotype D, across all seeds tested. This is a qualitative property of the model’s dynamics that does not depend on specific numerical choices.

The claim supported with comparable confidence is the sign and approximate magnitude of the association between synchronization and delirium: ρ(SI,declareddelirium)<0 in every replication, with a narrow spread (mean −0.797, SD 0.031) that is consistent across the main cohort, the ablation study, and the seed replications.

The claim that should be treated with caution is the magnitude of any specific result—individual delirium rates and the precise intermediate ordering of phenotypes B and C—because these depend on sample size and simulation length that have not been calibrated against patient data.

A further limitation concerns the nature of the causal mechanism. The prior sharpening loop was implemented in the model, not derived from it. The fact that the model reproduces the expected gradient and association when the mechanism is active provides evidence of internal coherence but does not constitute an independent validation of the causal hypothesis. The model is best understood as a coherent formalization of the predictive-synchronization hypothesis—one that produces the expected patterns when the hypothesized mechanism is active—rather than as a proof of the mechanism itself.

### 3.9. Toward an Active Inference Architecture of the ICU

Historically, the ICU room has been conceived primarily as a high-acuity clinical workspace: a space organized around equipment density, staff visibility and access, procedural efficiency, and infection prevention, rather than around the patient’s sensory, cognitive, or affective experience. This paper proposes a different conception. If delirium is the endpoint of a failure of predictive synchronization between patient and environment, then the sensory environment is not a background condition for clinical care—it is a primary therapeutic agent. The room does something. The question is whether what it does helps or harms.

The active inference framework makes this claim precise. Any artifact can be understood as a network of predictions about the agents who inhabit it [13]. A door handle predicts a grip; a hospital bed predicts a body; a light source predicts a circadian rhythm. These predictions are not metaphorical—they are structural. The artifact generates observations, and those observations either align with the occupant’s generative model or they do not. When they align, the occupant can predict what the environment will do next, and the environment—if it has a generative model—can predict what the occupant will do next. Synchronization is maintained. When they do not align, surprisal accumulates on both sides, and the system begins the descent toward the locked state we call delirium.

This reframing has a consequence that is not immediately obvious: the quality of an ICU room cannot be evaluated by measuring its physical properties alone. Lux levels, decibel readings, and temperature are necessary but not sufficient descriptors. What matters is whether the room’s sensory output is predictable from the patient’s current internal state, and whether the patient’s physiological responses are predictable from the room’s generative model. These are relational properties—they cannot be measured in the room without the patient, or in the patient without the room. They require a joint model of the coupling between the two, which is precisely what the present architecture provides.

Three design principles emerge from this reconceptualization:

#### 3.9.1. Personalization Is Not a Luxury—It Is a Structural Requirement

The ablation study demonstrates that a room agent without θ learning (M1) is structurally less capable of maintaining synchronization than one with it (M3), not because it tries less hard but because it uses the wrong model. A generic prior over patient parameters is wrong for every individual patient; it is only less wrong for some than others. The patients for whom it is most wrong are precisely the most vulnerable: those with dementia, sepsis, or frailty, whose true $\left(\theta_{\mathrm{cog}},\theta_{\mathrm{emo}}\right)$ are furthest from the population mean. A static sensory protocol applied uniformly across a heterogeneous patient population is therefore not merely suboptimal—it is systematically harmful to the patients who most need protection. The design implication is that ICU environments must be built around continuous inference about individual patients, not around fixed specifications derived from population averages.

#### 3.9.2. The Monitoring Layer Should Know When It Is Failing

The temporal-ordering analysis (Section 3.7) reveals that the environment-side model loses track of the patient before the patient loses track of the room: $S_{R\to P}$ rises first. This temporal asymmetry is not merely a monitoring insight—it is a design specification. It means that an environment-side inference layer maintaining a running generative model of the patient—whether realized through clinical staff supported by such a model or through an automated system—can detect its own loss of predictive accuracy before that failure becomes clinically visible, and can respond by changing the sensory output, alerting clinicians, or escalating the precision of its inference.

#### 3.9.3. Coherence Is Prior to Content

A common assumption in sensory environment design is that the relevant variable is the content of the stimulus: whether the light is warm or cool, whether the sound is music or noise, whether the schedule is diurnal or arrhythmic. The present model suggests that this assumption is incomplete. What matters at the level of mechanism is not primarily what the environment sends but how predictable what it sends is from the patient’s current state. Two environments that produce identical physical measurements may have radically different synchronization properties for different patients at different moments in their trajectory. A warm dim light is therapeutic for a patient whose generative model expects it and disruptive for a patient whose model cannot accommodate it. The design criterion is not stimulus content but mutual predictability—and mutual predictability is a dynamic, patient-specific, moment-by-moment property that no static protocol can fully capture.

These three design principles have direct and concrete implications for how delirium monitoring is integrated into everyday ICU practice.

In the current standard of care, delirium detection depends on periodic bedside assessments—typically two to three per day—using tools such as the CAM-ICU [1]. This approach is inherently retrospective: it captures the patient’s behavioral state at a discrete point in time, after the precision rigidity cycle may already be well advanced. The present model suggests a different logic. If $S_{R\to P}$—operationalized as the entropy of a continuously updated Bayesian model of the patient—rises before behavioral symptoms become clinically visible, then the relevant monitoring signal is not the patient’s behavior but the room’s predictive capacity. This reframes the clinical workflow in a precise way: instead of waiting for the patient to show signs of delirium, the system monitors whether the room has lost track of the patient, and generates an alarm when that loss of tracking exceeds a threshold.

Concretely, this would require three components integrated into the ICU infrastructure: first, a continuous physiological sensing layer—EEG, heart rate variability, actigraphy [41]—feeding real-time data into a Bayesian model of the patient’s hidden state $\left(\gamma_{\mathrm{cog}},\gamma_{\mathrm{emo}},\mathrm{circ}\right)$; second, a monitoring layer that tracks the entropy of that model at each cycle and computes the empirical analog of $S_{R\to P}$: when the entropy rises above a calibrated threshold, the room has lost predictive accuracy and an alarm is triggered; and third, a clinical response layer that specifies what happens when the alarm fires, including which clinician is notified, what environmental adjustment is recommended—a change in lighting, a reduction in acoustic stimulation, a reorientation intervention—and how the response is documented and fed back into the model to update the room’s belief about the patient’s parameters $\left(\theta_{\mathrm{cog}},\theta_{\mathrm{emo}}\right)$.

This workflow differs from the current practice in one fundamental respect: the alarm is generated by the environment’s failure to track the patient, not by the patient’s failure to pass a behavioral screening test. The clinical staff is therefore alerted at an earlier point in the causal chain—before the patient has entered the full precision rigidity regime—when environmental adjustment is still likely to be effective. This is not merely a monitoring improvement; it is a reconceptualization of the ICU room as an active participant in delirium prevention, one that continuously evaluates its own predictive capacity and triggers a clinical response when that capacity degrades. Whether this earlier alarm translates into better patient outcomes is an empirical question that the present model cannot answer but that a prospective study with simultaneous continuous physiological monitoring and standard clinical delirium assessment—using CAM-ICU positivity as the reference standard—could directly test.

## 4. Conclusions

This paper has proposed and implemented a two-agent active inference model of ICU delirium in which the predictive synchronization hypothesis is formalized as an explicit causal mechanism and tested for internal consistency across model variants, random seeds, and parameter configurations. The motivation is clinical and urgent: delirium affects 40–80% of mechanically ventilated patients, its hypoactive subtype is systematically missed by current screening tools, and the field lacks a mechanistic framework capable of specifying which environmental variables matter, why they matter, and for whom.

This paper proposes that the missing framework is predictive synchronization, and that the ICU environment—reconceptualized as an inferential layer that continuously models its occupant, whether realized through clinical staff or an automated monitoring system—is the missing therapeutic lever.

Three conclusions follow from the model. First, the ablation study identifies room inference as the architectural component necessary to reproduce the synchronization–delirium relationship: a non-inferring room produces ceiling delirium rates and a weak, unstable association, whereas any variant in which the room maintains a generative model of the patient yields a graded phenotypic response and a strong, stable association. The θ-learning and prior-sharpening mechanisms do not strengthen this association further; their role is to shape the phenotypic gradient—reducing ceiling effects in vulnerable phenotypes and amplifying the separation between them—which we interpret as evidence of internal coherence rather than independent validation. Second, $S_{R\to P}$—the room’s surprisal at the patient’s true state—differentiates clinical phenotypes substantially more than $S_{P\to R}$ and tends to cross the delirium threshold before it, consistent with the proposed causal sequence. This generates a falsifiable prediction: an entropy-based environment-side alarm should precede CAM-ICU positivity in prospective ICU monitoring data. Third, a room that has learned the patient’s latent parameters $\left(\theta_{\mathrm{cog}},\theta_{\mathrm{emo}}\right)$ generates less de-synchronization pressure and is less likely to trigger the precision rigidity cycle—making personalized environmental management not a clinical luxury but a structural requirement, and not a distant technological aspiration but a realizable next step given the sensing infrastructure already present in intensive care.

## Figures and Tables

**Table 1:** Clinical phenotypes: initial precision parameters and true latent parameters. Prevalence estimates from Inouye et al. [2] and Ely et al. [1].

ID	Phenotype	Prev.	$E{\left[\gamma_{\mathrm{cog}}\right]}_{0}$	$E{\left[\gamma_{\mathrm{emo}}\right]}_{0}$	True $\left(\theta_{\mathrm{cog}},\;\theta_{\mathrm{emo}}\right)$
A	Young, healthy	15%	2.05	0.70	(1.00, 1.00)
B	Elderly, frail	40%	1.65	1.15	(0.65, 1.30)
C	Septic, ventilated	30%	1.40	1.80	(0.50, 1.70)
D	Dementia/MCI	15%	1.05	1.60	(0.35, 1.45)

**Table 2:** Model comparison across four variants (n=12 per phenotype, 150 cycles, seeds {1,7,42}). Del% values are mean ± SD across seeds. ρ: Spearman rank correlation between mean synchronization index and declared delirium (distribution-free; replaces the Pearson *r* used previously, which is inappropriate for this thresholded relationship). Grad: fraction of seeds in which the phenotypic gradient A<B,C,D is preserved.

Model	A Del%	B Del%	C Del%	D Del%	ρ (Mean ± SD)	Grad
M0: Random room	89±10	86±8	94±8	94±4	−0.43±0.10	1/3
M1: No θ learning	19±10	58±12	53±14	50±7	−0.84±0.03	2/3
M2: No causal feedback	17±7	42±18	47±4	33±18	−0.78±0.06	1/3
M3: Full model	17±7	42±18	53±8	39±14	−0.80±0.07	2/3

**Table 3:** Summary results by phenotype (120 patients, 200 decision cycles, seed 42, full model with prior sharpening active). Surprisal and learning values are mean ± SD across the 30 patients within each phenotype (sample SD, ddof=1). $S_{R\to P}$: room surprisal at the patient’s true state. $S_{P\to R}$: patient surprisal at room observations (true symmetric metric, Equation (2)). *ℓ*: θ learning progress. Delirium is the binary declared-delirium outcome (Definition 2). The within-phenotype SD of $S_{R\to P}$ is comparable to or larger than the between-phenotype spread of its mean, indicating that the phenotypic ordering is a difference in means rather than a separation at the individual-patient level.

Phenotype	Del%	SI	$S_{R\to P}$	$S_{P\to R}$	*ℓ*
A: Young, healthy	26.7	0.54±0.10	2.75±1.58	2.97±0.34	0.09±0.05
B: Elderly, frail	40.0	0.51±0.12	2.93±1.01	3.05±0.43	0.10±0.06
C: Septic, ventilated	53.3	0.48±0.13	3.78±2.96	3.13±0.46	0.09±0.05
D: Dementia / MCI	53.3	0.46±0.13	3.37±1.18	3.18±0.56	0.07±0.05
Overall: 120 patients, 52 delirious (43%).					
Logistic P(delirium)∼ mean SI: $\beta_{1}=-45.56$ (95% CI [−64.52,−26.60]), AUC =0.97.					
Spearman ρ(SI,delirium)=−0.81 (Pearson r=−0.78, for comparison).					

**Table 4:** Robustness of key results across 5 independent random seeds (n=20 patients per phenotype, 200 decision cycles, prior sharpening active). Values are delirium rates (%) except where indicated. ρ is the Spearman rank correlation between the mean synchronization index and the binary declared-delirium outcome.

Metric	Mean	SD	Min	Max
A: Young, healthy—Del%	38.0	9.8	25	55
B: Elderly, frail—Del%	51.0	5.8	45	60
C: Septic, ventilated—Del%	51.0	8.6	40	65
D: Dementia/MCI—Del%	63.0	8.1	50	75
Overall delirium rate (%)	50.8	2.8	46	54
ρ(SI,declareddelirium)	−0.797	0.031	−0.852	−0.763

## Data Availability

Data is contained within the article.
